# Supplementary material for: The oral intake of specific Bioactive Collagen Peptides (BCP) improves gait and quality of life in canine osteoarthritis patients—A translational large animal model for a nutritional therapy option
Source: PLoS One. 2024 Sep 19;19(9):e0308378. doi: 10.1371/journal.pone.0308378 (PMC11412516; doi:10.1371/journal.pone.0308378)
Supplement: S1 Table — (DOCX) [file pone.0308378.s001.docx]

**Appendix Table 1:** Absolute difference (∆) in gait analysis parameters of affected and unaffected limbs between the second (T12) and first (T0) examination, grouped by supplementation (absolute difference; median [min; max]).

|  | ∆ PVF  [% BW] | ∆VI  [% BW * s] | ∆DSP  [% of step] |
| --- | --- | --- | --- |
|  | PLA | | |
| Unaffected limb  (n = 27) | 0.99  [-18.8; 9.6] | 0.07  [-6.5; 8.4] | 0.00  [-6.3; 7.5] |
| p-value | 0.952 | 0.904 | 0.899 |
| Affected limb  (n = 9) | 0.05  [-9.0; 18.8] | -0.18  [-4.3; 3.4] | 0.00  [-5.5; 8.5] |
| p-value | 0.953 | 0.515 | 0.674 |
|  | BCP | | |
| Unaffected limb  (n = 32) | -0.53  [-11.3; 13.3] | -0.07  [-8.4; 3.9] | 0.67  [-12.3; 6.1] |
| p-value | 0.295 | 0.340 | 0.724 |
| Affected limb  (n = 12) | 2.74  [-14.3; 20.0] | 1.05  [-3.0; 8.0] | 1.75  [-11.8; 7.5] |
| p-value | 0.071 | 0.015 | 0.410 |
|  | n3FA | | |
| Unaffected limb  (n = 33) | -0.90  [-7.8; 14.9] | -0.18  [-4.3; 6.4] | -0.50  [-10.5; 11.0] |
| p-value | 0.081 | 0.598 | 0.674 |
| Affected limb  (n = 11) | -0.71  [-17.7; 10.0] | 0.45  [-1.9; 3.6] | 0.00  [-3.5; 8.3] |
| p-value | 0.594 | 0.477 | 0.919 |

PVF = peak vertical force; VI = vertical impulse; DSP = duration of stance phase; ∆ = difference
