# Supplementary material for: The oral intake of specific Bioactive Collagen Peptides (BCP) improves gait and quality of life in canine osteoarthritis patients—A translational large animal model for a nutritional therapy option
Source: PLoS One. 2024 Sep 19;19(9):e0308378. doi: 10.1371/journal.pone.0308378 (PMC11412516; doi:10.1371/journal.pone.0308378)
Supplement: S2 Table — (DOCX) [file pone.0308378.s002.docx]

**Appendix Table 2:** Relative difference [%] in gait analysis parameters of affected and unaffected limbs between the first (T0) and second (T12) examination (%; median [min; max]).

|  | RPD in PVF | RPD in VI | RPD in DSP |
| --- | --- | --- | --- |
|  | PLA | | |
| Unaffected limb  (n = 27) | 2.01  [-24.7; 21.6] | 0.61  [-32.5; 46.8] | -0.34  [-9.3; 10.1] |
| p-value | 0.302 | 0.683 | 0.501 |
| Affected limb  (n = 9) | -2.14  [-18.7; 21.1] | -10.15  [-27.06; 20.87] | -2.22  [-7.6; 12.2] |
| p-value | 0.859 | 0.110 | 0.779 |
|  | BCP | | |
| Unaffected limb  (n = 32) | -0.65  [-16.5; 17.8] | -1.12  [-39.5; 51.3] | -0.52  [-18.4; 9.2] |
| p-value | 0.379 | 0.350 | 0.597 |
| Affected limb  (n = 12) | 9.47  [-2.0; 29.6] | 2.14  [-27.7; 82.5] | -0.93  [-16.1; 14.4] |
| p-value | 0.005 | 0.583 | 0.937 |
|  | n3FA | | |
| Unaffected limb  (n = 33) | -1.60  [-12.8; 27.2] | -0.91  [-16.1; 31.2] | -1.43  [-14.7; 8.0] |
| p-value | 0.033 | 0.993 | 0.088 |
| Affected limb  (n = 11) | 0.18  [-6.3; 32.8] | 3.93  [-15.2; 25.6] | -2.05  [-8.3; 5.5] |
| p-value | 0.424 | 0.594 | 0.333 |

PVF = peak vertical force; VI = vertical impulse; DSP = duration of stance phase; ∆ = difference
