# Supplementary material for: The oral intake of specific Bioactive Collagen Peptides (BCP) improves gait and quality of life in canine osteoarthritis patients—A translational large animal model for a nutritional therapy option
Source: PLoS One. 2024 Sep 19;19(9):e0308378. doi: 10.1371/journal.pone.0308378 (PMC11412516; doi:10.1371/journal.pone.0308378)
Supplement: S3 Table — (DOCX) [file pone.0308378.s003.docx]

**Appendix Table 3:** Probability of error (p) for the symmetry indices of peak vertical force and vertical impulse concerning the threshold value of 10 (SI PVF) and 9 (SI VI), respectively.

|  | SI PVF | SI VI |
| --- | --- | --- |
|  | PLA | |
| T0 | 0.544 | 0.173 |
| T12 | 0.678 | 0.515 |
|  | BCP | |
| T0 | 0.508 | 0.638 |
| T12 | 0.041 | 0.937 |
|  | n3FA | |
| T0 | 0.875 | 1.000 |
| T12 | 0.117 | 0.480 |

PVF = peak vertical force; VI = vertical impulse
